# Mesenchymal stem cells augmentation for surgical procedures in patients with symptomatic chondral defects of the knee: a systematic review

**DOI:** 10.1186/s13018-022-03311-1

**Published:** 2022-09-14

**Authors:** Migliorini Filippo, Mangiavini Laura, Giorgino Riccardo, Vismara Valeria, Jörg Eschweiler, Nicola Maffulli

**Affiliations:** 1 Department of Orthopedic, Trauma, and Reconstructive Surgery, RWTH Aachen University Hospital, Pauwelsstraße 30, 52074 Aachen, Germany; 2 IRCCS Istituto Ortopedico Galeazzi, Milan, Italy; 3 Department of Biomedical Sciences for Health, University of Milan, Milan, Italy; 4 Residency Program in Orthopedics and Traumatology, University of Milan, Milan, Italy; 5 Department of Medicine, Surgery and Dentistry, University of Salerno, Via S. Allende, 84081 Baronissi, SA Italy; 6 Barts and the London School of Medicine and Dentistry, Centre for Sports and Exercise Medicine, Mile End Hospital, Queen Mary University of London, 275 Bancroft Road, London, E1 4DG England; 7 School of Pharmacy and Bioengineering, Keele University Faculty of Medicine, Thornburrow Drive, Stoke on Trent, England

**Keywords:** Knee, Chondral defects, Mesenchymal stem cells, MSCs

## Abstract

**Background:**

The efficacy and safety profile of mesenchymal stem cells (MSCs) augmentation in chondral procedures are controversial. This systematic review updated the current evidence on MSCs augmentation for chondral procedures in patients with symptomatic chondral defects of the knee.

**Methods:**

This study followed the PRISMA guidelines. The literature search was updated in August 2022. Two independent authors accessed PubMed, Google scholar, Embase, and Scopus. No additional filters or time constrains were used for the search. A cross reference of the bibliographies was also performed. All the clinical studies investigating surgical procedures for chondral defects of the knee augmented with MSCs were accessed. Defects of both tibiofemoral and patellofemoral joints were included. The following patient reported outcomes measures (PROMs) were retrieved at baseline and last follow-up: Visual Analogic Scale (VAS), Tegner Activity Scale, Lysholm Knee Scoring System, International Knee Documentation Committee (IKDC). Return to daily activities and data on hypertrophy, failure, revision surgery were also collected. Failures were defined as the recurrence of symptoms attributable to the index procedure. Revisions were defined as any reoperation at the site of the index procedure.

**Results:**

A total of 15 clinical studies (411 procedures) were included. Patients returned to their prior sport activity at 2.8 ± 0.4 months. All the PROMs improved at last follow-up: Tegner (*P* = 0.0002), Lysholm (*P* < 0.0001), the IKDC (*P* < 0.0001), VAS (*P* < 0.0001). At a mean of 30.1 ± 13.9 months, 3.1% (2 of 65 patients) reported graft hypertrophy, 3.2% (2 of 63) were considered failures. No surgical revision procedures were reported. Given the lack of available quantitative data for inclusion, a formal comparison of surgical procedures was not conducted.

**Conclusion:**

MSCs augmentation in selected chondral procedures could be effective, with a low rate of complications. Further investigations are required to overcome the current limitations to allow the clinical translation of MSCs in regenerative medicine.

## Introduction

Chondral defects of the knee are common. Most are traumatic, and involve the medial femoral condyle [1, 2]. Given the poor healing capability of the articular cartilage, these defects have limited chance to heal [3, 4]. Chondral defects may cause persistent pain, reducing the quality of life, and sport participation, and may predispose to early onset osteoarthritis [1, 3]. Microfractures have been proposed for lesions up to 2.5 cm^2^ [5–7]. For larger defects, autologous chondrocyte implantation (ACI) has been proposed [3, 8]. ACI is a two-step surgical procedure which necessitates chondral harvesting from a non-weight-bearing area of the knee and external chondrocytes expansion in a dedicated laboratory [9, 10]. Autologous matrix-induced chondrogenesis (AMIC) is also widely performed to regenerate chondral defects [11–13]. AMIC combines microfractures with an acellular membrane scaffold in a single-session surgery [13, 14]. AMIC exploits the regenerative potential of the mesenchymal stem cells arising from the subchondral bone [15, 16]. These procedures have been augmented with MSCs to enhance their regenerative potential. Mesenchymal stem cells (MSCs) derived from bone marrow aspirate concentrate (BMAC) or adipose tissue have also been used to enhance chondral procedures with promising results [17–21]. The use of MSCs seeded on a bio-degradable scaffold, with or without growth factors augmentation, has shown promise in animal and clinical studies [22–27]. MSCs are able to maintain multipotency during culture expansion [27], and to differentiate into chondrocytes [28]. Moreover, these cells are characterized by self-renewal capacity, plasticity and the ability to migrate toward injury sites, where they demonstrate trophic effects and immunomodulatory potential, inhibiting T and B cell proliferation and NK cell activation [29–31]. The current literature evidences a high ratio of preclinical to clinical studies on this topic, suggesting that we are in a transition phase to clinical application in human. Thus, this systematic review updated the current evidence on MSCs application in chondral defects of the knee. The outcome of interest was to assess efficacy and safety of MSCs augmentation for chondral procedures in patients with symptomatic chondral defects of the knee.

## Methods

### Search strategy

This systematic review was conducted according to the Preferred Reporting Items for Systematic Reviews and Meta-Analyses: the PRISMA guidelines [32]. The PICOT algorithm was preliminary pointed out:

- P (Population): symptomatic knee chondral defects;
- I (Intervention): MSCs augmentation of surgical procedures for chondral defects of the knee;
- C (Comparison): efficacy and safety;
- O (Outcomes): PROMs, return to sport;
- T (Timing): minimum 12 months follow-up.

### Data source and extraction

Two authors (F.M. and R.G.) independently performed the literature search. The last update of the search was conducted in August 2022 accessing PubMed, Google scholar, Embase, and Scopus. The following keywords were used in combination using the Boolean operator AND/OR: *knee* OR/AND *chondral defects* AND *(focal* OR *surgery* OR *pain* OR *sports* OR *surgery* OR *therapy* OR *management* OR *arthroscopy* OR *augmentation* OR *enhance* OR *application)* AND *(ACI* OR *autologous chondrocyte implantation* OR *matrix-induced* OR *periosteum* OR *membrane* OR *chondral* OR *collagen* OR *stem cells* OR *bone marrow* OR *adipose* OR *peripheral* OR *blood* OR *concentrate* OR *mesenchymal)* AND *(visual analogic scale* OR *PROM* OR *patient reported outcome measures* OR *outcome* OR *revision* OR *failure).* No additional filters or time constrains were used for the search. The same authors independently screened the resulting articles. If title and abstract matched the topic, the full-text article was accessed. A cross reference of the bibliographies was also performed. Disagreements were debated and solved by a third author (M.N.).

### Eligibility criteria

All the clinical studies investigating surgical procedures for chondral defects of the knee augmented with MSCs were accessed. According to the authors language capabilities, articles in English, German, Italian, French and Spanish were eligible. Level I to IV of evidence, according to Oxford Center of Evidence-Based Medicine [33], were considered. Studies that combined multiple chondral strategies or MSCs were not eligible, nor were those addressing multiple chondral defects. We included only studies that enhanced chondral procedures with MSCs. Studies which employed innovative hydrogel were not eligible, nor were those including synthetic scaffolds/polymers [34–39]. Studies were included irrespectively of the size, location (tibiofemoral and patellofemoral), and depth (chondral and osteochondral) of the chondral defect, the cell delivery methods used, and the concomitant procedures. Moreover, studies were included irrespective of the sources of MSCs (adipose, bone marrow, synovial, peripheral blood), culture, expansion, and implantation modalities. Only articles reporting quantitative data under the outcomes of interest were considered.

### Data extraction

Two authors (F.M. and R.G.) independently performed data extraction. Study generalities (author, year, journal, type of study, length of the follow-up) and patients baseline characteristics (number of procedures, mean BMI, age, and defect size, mean duration of the symptoms, percentage of female and right side) were collected. The following data were retrieved at baseline and at last follow-up: visual analog scale (VAS), Lysholm Knee Scoring Scale [40], Tegner Activity Scale [41] and International Knee Documentation Committee (IKDC) [42]. Data concerning patients return to daily activity were also collected. Further, data on complications were also collected: hypertrophy, failure, arthroplasty, revision surgery. Failures were defined as the recurrence of symptoms attributable to the index procedure. Revisions were defined as any reoperation at the site of the index procedure.

### Methodology quality assessment

The methodological quality assessment was performed by one author (R.G.) using the risk of bias graph tool of the Review Manager Software (The Nordic Cochrane Collaboration, Copenhagen). This tool evaluated the major risk of bias of each included study. The following risks of bias were evaluated: selection (random sequence generation and the allocation concealment), detection (assessor blinding), attrition, reporting, and other (additional risk of bias) source of bias. Disagreements were solved by a third senior author (N. M.). The risk of bias was evaluated in percentage as low (green), high (red), or unclear (yellow).

### Statistical analysis

The statistical analyses were performed using the IBM SPSS software (version 25). To evaluate possible differences of continuous variables (post vs pre-operative data), the mean difference (MD) effect measure was adopted. The t-test was performed, with *P* values of < 0.05 considered statistically significant. The confidence interval (CI) was set at 95% in all the comparisons. For binary data (rate of complication), the number of events reported in each single study was evaluated.

## Results

### Search result

**Fig. 1:**
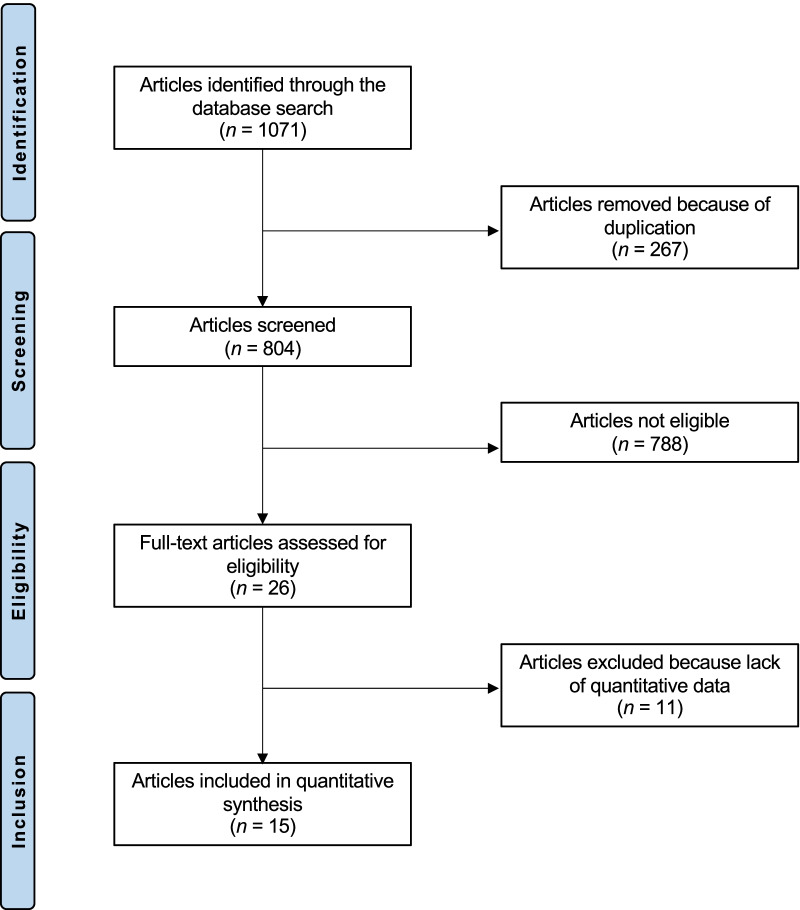
Flowchart of the literature search

The literature search resulted in 1071 articles. Of them, 267 were duplicates. A further 778 studies were not eligible: study design (*N* = 231), not focused on knee (*N* = 101), not focused on chondral defects management (*N* = 199), not enhanced with MSCs (*N* = 221), combined treatment (*N* = 3), type of surgical intervention (*N* = 20), language limitation (*N* = 2), uncertain results (*N* = 1). A further 11 studies were excluded because lacking of quantitative data under the outcomes of interest. This left 15 clinical investigations for the present study: two RCTs, seven prospective and six retrospective studies. The literature search results are shown in Fig. 1.

### Methodological quality assessment

**Fig. 2:**
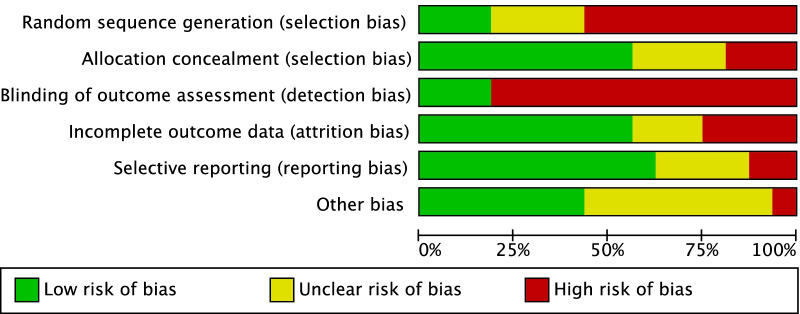
Cochrane risk of bias tool. Each risk of bias (selection, detection, attrition, reporting, and other bias) was evaluated in percentage as low (green), high (red), or unclear (yellow)

The risk of selection, detection, attrition, reporting, and other bias were evaluated. The risk of selection bias was moderate. Indeed, 40% (6/15) of the included studies were retrospective, and 87% (13/15) did not perform randomization. Assessor blinding was seldom performed, increasing the risk of performance bias. The risk of reporting and detection biases was low-moderate, as was the risk of the risk of other biases. Concluding, the methodological assessment showed an acceptable quality (Fig. 2).

### Patient demographics

Data from 411 procedures were retrieved. The mean duration of symptoms before the procedure was 7.3 ± 6.9 months. The mean follow-up was 30.1 ± 13.9 months. The mean age of the patients was 36.3 ± 9.8 years, the mean BMI was 25.2 ± 1.4 kg/m^2^. 41% (169 of 411 patients) were women, while 52% (214 of 411) of patients the defect was located on the right side. The mean defect size was 4.6 ± 2.3 cm^2^ (Table 1).

**Table 1:** Generalities and patient baseline of the included studies

References	Journal	Design	Patients (*n*)	Follow-up (months)	Female (%)	Mean age	Type of augmentation
Akgun et al. [47]	*Arch Orthop Trauma Surg*	RCT	7	24	57	32	MSCs
			7	24	57	33	Control group
Buda et al. [2]	*J Bone Joint Surg Am*	Prospective	20	29	40		BM-MSCs
Buda et al. [54]	*Europ J Orthop Surg Traumatol*	Retrospective	28	48	43	38	BMAC
De windt et al. [20]	*Stem Cells*	Prospective	10	12	20	26	Allogenic MSCs
De windt et al. [50]	*Stem Cells*	Prospective	35	18	31	30	Allogenic MSCs
Enea et al. [43]	*Knee*	Retrospective	9	22	45	48	BMAC
Enea et al. [57]	*Knee*	Retrospective	9	29	44	43	BMAC
Gobbi et al. [18]	*Cartilage*	Prospective	15	24	33	48	BMAC
Gobbi et al. [19]	*Am J Sports Med*	Prospective	25	41	36	47	BMAC
Gobbi et al. [44]	*Knee Surg Sports Traumatol Arthrosc*	Prospective	20	48		50	BMAC
Haleem et al. [25]	*Cartilage*	Retrospective	5	12	20	25	BM-MSCs
Koh et al. [48]	*Arthroscopy*	RCT	40	27	65	38	A-MSCs
			40	27	60	39	Control group
Nejadnik et al. [45]	*Am J Sports Med*	Retrospective	36	24	50	43	Control group
			36	24	44	44	BM-MSCs
Skowronski et al. [58]	*Ortop Traumatol Rehabil*	Prospective	21	60	42	26	Control group
			25	60	44	26	Blood MSC
Teo et al. [46]	*Clin Orthop Relat Res*	Retrospective	20	24	20	17	Control group
			3	24	20		BM-MSC

BMAC—bone marrow aspirate concentrate; TT—tibial tubercle; A-MSCs—adipose derived MSCs; BM-MSCs—bone marrow derived MSCs; Coll—collagen; RCT—randomized controlled trial

### Outcomes of interest

Patients returned to their prior sport activity at a mean of 2.8 ± 0.4 months. At a mean of 30.1 ± 13.9 months, the Tegner scale increased of 2.8 points (*P* = 0.0002), the Lysholm score of 32.9 points (*P* < 0.0001), the IKDC of 36.8 (P < 0.0001). The VAS (0–10) decreased of − 4.6 (< 0.0001). The results of PROMs are shown in greater detail in Table 2.

**Table 2:** Results of PROMs

Endpoint	Pre-operative	Post-operative	MD	P
VAS	5.7 ± 1.9	1.1 ± 0.6	− 4.6	< 0.0001
Tegner	2.0 ± 0.7	4.8 ± 0.9	2.8	0.0002
Lysholm	53.3 ± 8.9	86.2 ± 6.9	32.9	< 0.0001
IKDC	46.0 ± 6.4	82.8 ± 5.2	36.8	< 0.0001

### Complications

Few studies reported data on complications. 3.1% (2 of 65 patients) developed graft hypertrophy. 3.2% (2 of 63 patients) experienced persistent symptomatic pain at the site of the index procedure and were considered failure. No revision procedure was reported.

## Discussion

According to the main findings of the present study, chondral procedures augmented with MSCs demonstrated efficacy and feasibility. Irrespective of the surgical procedure, a statistically significant improvement in PROMs was evidenced, with return to previous sporting activities within three months.

MSCs are able to maintain multipotency during culture expansion [27], and to differentiate into chondrocytes [28]. Moreover, these cells are characterized by self-renewal capacity, plasticity and the ability to migrate toward injury sites, where they demonstrate trophic effects and immunomodulatory potential, inhibiting T and B cell proliferation and NK cell activation [29–31]. Animal studies have demonstrated that, compared to the other techniques, chondral procedures augmented with MSCs give rise to a greater thickness, a smoother surface and greater integration of the neo-cartilage with the surrounding native cartilage, probably from improved type II collagen content and orientation [27]. The properties and differentiation abilities of MSCs may differ according to their source [29]. Several methods are used to obtain and deliver MSCs. Bone marrow (BM), synovial, and adipose tissues are well established sources of MSCs. BM-MSCs are commonly harvested from the posterior iliac crest. After harvesting, BM-MSCs are centrifuged to remove erythrocytes and plasma cells, and subsequently seeded in either a hyaluronic acid membrane [2, 43, 44], or collagen type I/III matrix [18, 19]. In other protocols, MSC from BMA are first cultured and later inserted into the defect [25, 45, 46]. Although the MSC yield per unit is not high [31], they can be easily collected, and have a good osteogenic and chondrogenic potential [29]. On the other hand, the differentiation ability of BM-MSCs seems to decrease with age, the harvesting procedure can be painful, and the amount of de novo hyaline cartilage can be limited [28]. In the present study, only one article documented the successful use of synovium derived MSCs [47]. The harvesting technique is less painful than BM harvesting since the synovial tissue is harvested arthroscopically from the supracondylar region of the femoral condyle of the operated knee, to be later minced, digested, centrifuged, and cultured. These amplified MSCs are then suspended within a culture medium and pipetted onto a collagen membrane, thereby giving rise to a membrane cell construct, known as matrix-induced autologous mesenchymal cell implantation (mAMI). Synovial derived MSCs demonstrate a greater chondrogenic and less osteogenic potential than those deriving from bone marrow [29, 47]. Furthermore, they seem to maintain greater differentiation potential regardless of age, yield greater amount of MSC per unit, and decrease total costs and culture time [29, 30]. Other authors used adipose derived MSCs from the subcutaneous adipose buttock tissue [48]. Lipoaspirate is collected using syringe suction, the stromal vascular structure containing MSCs is separated from mature adipocytes by centrifugation, and later suspended in platelet rich plasma. Adipose-MSCs can be isolated in large quantity, are easily available, possess anti-inflammatory properties and have a similar proliferative profile to BM-MSCs, and do not seem to be influenced by age [29, 49]. On the other hand, they seem to have a lower chondrogenic potential compared to BM-MSCs and synovial MSCs [29].

Allogenic MSCs have also been used [20, 50]. A mix of allogenic MSCs, autologous chondrons, derived from the patient’s recycled cartilage tissue, and fibrin glue has been delivered directly at the lesion site in a single surgical session, avoiding donor site morbidity. Despite the allogenicity of these cells, the authors reported no adverse immune response and a good tissue integration, comparable, if not superior, to ACI procedures [20, 50]. Other authors, instead, used autologous BMAC for augmentation. Harvested autologous BM from the posterior iliac crest undergoes centrifugation to concentrate pluripotent mesenchymal stem cells, growth factors, platelets, and white blood cells [51]. BMAC can be later used on its own and injected over the chondral defect, or can be combined with either microfractures [43], chondral grafting, or biphasic scaffolds [18, 19, 44, 51]. Although these procedures have been associated with low complication rates [51], it is still not clear whether they really enhance the regeneration of hyaline-like cartilage tissue [52, 53]. Moreover, the composition of BMAC varies widely among individuals [51]; therefore, clinical results may be different according to patient demographics, especially when younger subjects are compared to older individuals [52].

Cell delivery can be performed through different methodologies with a variable degree of invasiveness, from arthroscopy [2, 43, 48] to mini arthrotomy [18–20, 50], or formal arthrotomy [25, 45, 46, 54]. Irrespective to the type of cell therapy, the use of a periosteal flap mandates a parapatellar arthrotomy, leading to increased donor site morbidity and greater invasiveness. Better efficacy, lesser donor site morbidity, and fewer complications are achieved when the periosteal flap is substituted with a bio-degradable scaffold, either collagen type I/III or hyaluronan, to fill the defect. These membrane cell constructs can be delivered either through mini arthrotomy [18, 19, 44, 47] or arthroscopically [43]. A mini arthrotomy is also employed when recycled cartilage is needed to obtain autologous chondrons [20, 50].

We are aware that the present study presents several limitations. The retrospective nature of most of the included studies negatively impacted the reliability of the results, leading to high risk of selection bias. The sample size was small in most studies, with 27% (4/15) reporting data on less than 10 patients. In only 27% (4/15) of studies was the follow-up longer than 24 months. Hence, long term complications have not been investigated. In addition, the size, location (tibiofemoral and patellofemoral), and depth (chondral and osteochondral) of the chondral defects, and the cell delivery methods used are heterogeneous, and preclude statistical analysis [27, 29, 49, 51, 52, 55, 56]. The type of chondral procedure was heterogeneous in the included studies, which also represents an important source of bias. Most authors used a collagen or hyaluronic membrane to stabilize the blood coat within the knee cavity. Whether the nature of the membrane used influence the outcome is unclear, and future investigations are required. Histological analysis was seldom performed, representing another limitation. Moreover, in most of the included studies concomitant procedures, such as meniscectomy, synovectomy, anterior cruciate ligament repair, and high tibial osteotomy, were performed [2, 18, 19, 43–48]. Several MSCs sources (adipose, bone marrow, synovial, peripheral blood), culture, expansion, and implantation modalities have been described, but seldom compared to one another. Thus, it is difficult to understand whether a given technique is superior to another. Further studies should investigate the optimal cell source and dosage [27]. Between studies, heterogeneity in the eligibility criteria and timing of assessment of outcome was evident. Patient selection was not standardized. The role of age on the regenerative potential of MSCs and autologous chondrocytes is still unclear, and comparative investigations should be undertaken. Imaging and histological analyses were undertaken at different time intervals between the studies, representing a further limitation. Further investigations are required to establish the superiority of MSCs augmentation over the isolated procedures in a clinical setting. Given these limitations, results from the present study must be interpreted with caution.

## Conclusion

MSCs augmentation in selected chondral procedures could be effective, with a low rate of complication. Further investigations are required to overcome current limitations to the clinical translation of MSCs in the regenerative medicine.

## Data Availability

No new data were generated or analyzed in support of this review.

## Abbreviations

Microfractures: Microfractures
ACI: Autologous chondrocyte implantation
AMIC: Autologous matrix-induced chondrogenesis
BMAC: Bone marrow aspirate concentrate
MSCs: Mesenchymal stem cells
BM: Bone marrow
mAMI: Matrix-induced autologous mesenchymal cell implantation
PROMs: Patient reported outcome measures
